# RETRACTIONS: Isolation, Identification, Characterization, and Plasmid Profile of Urinary Tract Infectious *Escherichia coli* from Clinical Samples

**DOI:** 10.1155/ecam/9754209

**Published:** 2026-02-10

**Authors:** Evidence-Based Complementary and Alternative Medicine

RETRACTION: S. Thangavelu, R. Dhandapani, A. Arulprakasam, et al., “Isolation, Identification, Characterization, and Plasmid Profile of Urinary Tract Infectious *Escherichia coli* from Clinical Samples,” *Evidence-Based Complementary and Alternative Medicine*, 2022, 7234586, https://doi.org/10.1155/2022/7234586.

The above article, published online on 20 March 2022 in Wiley Online Library (https://wileyonlinelibrary.com), has been retracted by John Wiley & Sons Ltd.

This article has been retracted following an investigation undertaken by the publisher. This investigation has uncovered evidence of one or more of the following indicators of systematic manipulation of the publication process:

1. Discrepancies in scope
2. Discrepancies in the description of the research reported
3. Discrepancies between the availability of data and the research described
4. Inappropriate citations
5. Incoherent, meaningless, and/or irrelevant content included in the article
6. Manipulated or compromised peer review

The presence of these indicators undermines our confidence in the integrity of the article’s content and we cannot, therefore, vouch for its reliability. Please note that this notice is intended solely to alert readers that the content of this article is unreliable. We have not investigated whether authors were aware of or involved in the systematic manipulation of the publication process.

K. Durairaj claims they were not aware of the submission and did not contribute to the article. The remaining authors disagree with the retraction.

